# Fat-free/lean body mass in children with insulin resistance or metabolic syndrome: a systematic review and meta-analysis

**DOI:** 10.1186/s12887-021-03041-z

**Published:** 2022-01-22

**Authors:** Diana Paola Córdoba-Rodríguez, Iris Iglesia, Alejandro Gomez-Bruton, Gerardo Rodríguez, José Antonio Casajús, Hernan Morales-Devia, Luis A. Moreno

**Affiliations:** 1 Departamento de Nutrición y Bioquímica, Facultad de Ciencias, Pontificia Universidad Javeriana, Bogotá, DC Colombia; 2 Growth, Exercise, Nutrition and Development (GENUD) Research Group, Universidad de Zaragoza, Zaragoza, Spain; 3 Instituto Agroalimentario de Aragón (IA2), Instituto de Investigación Sanitaria Aragón (IIS Aragón), Zaragoza, Spain; 4 Red de Salud Materno Infantil y del Desarrollo (SAMID), Instituto de Salud Carlos III, Madrid, Spain; 5 Faculty of Health and Sport Sciences (FCSD), Department of Physiatry and Nursing, University of Zaragoza, Zaragoza, Spain; 6 Centro de Investigación Biomédica en Red de Fisiopatología de la Obesidad y Nutrición (CIBERObn), Instituto de Salud Carlos III, Madrid, Spain; 7 Departamento de Pediatría, Universidad de Zaragoza, Zaragoza, Spain; 8 Biblioteca General Alfonso Borrero Cabal, Pontificia Universidad Javeriana, Bogotá, Colombia

**Keywords:** Body composition, Insulin resistance, Metabolic syndrome, Infant, Child, Adolescent

## Abstract

**Background:**

Lean / Fat Free Body Mass (LBM) is metabolically involved in active processes such as resting energy expenditure, glucose uptake, and myokine secretion. Nonetheless, its association with insulin sensitivity / resistance / glucose tolerance and metabolic syndrome remains unclear in childhood.

**Methods:**

The current investigation aimed to examine the differences in fat-free mass /lean body mass according to the presence of insulin sensitivity/insulin resistance/glucose tolerance/metabolic syndrome in children.

A systematic search was carried out in Medline/PubMed, Embase, Scopus, Web of Science, and SciELO, covering the period from each database’s respective start to 21 June 2021. Two researchers evaluated 7111 studies according to the inclusion criteria: original human studies, written in English or Spanish, evaluating fat-free mass/lean body mass in children and adolescents including both with and without insulin sensitivity/insulin resistance /glucose tolerance and metabolic syndrome and reported the differences between them in terms of fat free mass/lean body mass.

The results of the studies were combined with insulin sensitivity, insulin, resistance, glucose tolerance and metabolic syndrome. The standardized mean difference (SMD) in each study was calculated and combined using the random-effects model. Heterogeneity between studies was tested using the index of heterogeneity (I^2^), leave-one-out sensitivity analyses were performed, and publication bias was assessed using the Egger and Begg tests.

**Results:**

Finally, 15 studies which compared groups defined according to different glucose homeostasis criteria or metabolic syndrome out of 103 eligible studies were included in this systematic review and 12 studies in the meta-analysis. Meta-analysis showed lower fat-free mass/lean body mass percentage in participants with insulin resistance/glucose tolerance/metabolic syndrome (SMD -0.47; 95% CI, − 0.62 to − 0.32) while in mass units (kg), higher values were found in the same group (SMD, 1.01; 95% CI, 0.43 to 1.60).

**Conclusions:**

Our results identified lower values of fat-free mass/lean body mass (%) in children and adolescents with insulin resistance/glucose tolerance/metabolic syndrome and higher values of fat-free mass/lean body mass when these are expressed in kg. The evidence of the impact of lean mass on children’s glucose homeostasis or metabolic syndrome is limited, so future studies research should focus on explaining the effect of fat-free mass/lean body mass on different metabolic outcomes. Moreover, it may be interesting to evaluate the quality (muscle density) or functional (muscle strength) outcomes in addition to both absolute (kg) and relative (%) values in future studies.

The systematic review was prospectively registered at PROSPERO (registration number CRD42019124734; available at: http://www.crd.york.ac.uk/prospero [accessed: 05 April 2019]).

**Supplementary Information:**

The online version contains supplementary material available at 10.1186/s12887-021-03041-z.

## Key notes

- Our findings indicate a lower percentage of fat-free/lean body mass in participants with insulin resistance/glucose tolerance/metabolic syndrome, while higher values were found when expressed in kg.
- The heterogeneity between the studies, should be considered when analyzing the results.
- The evidence on the impact of lean mass on glucose homeostasis in children is limited.

## Background

Insulin resistance (IR) is defined as the reduction of the tissue’s response to insulin action, and it is the opposite of insulin sensitivity (IS) [1]. Insulin resistance is significant in public health. Its persistence over time and its tendency to progress clinically are the first stages of the development of Type 2 diabetes [2]. Currently, the fluctuations of IR prevalence in children and adolescents range from 2.2% in those with a healthy weight to 10.8% in those with obesity [3]. Insulin resistance is recognized as a central component of metabolic syndrome (MetS) [4], characterized by central obesity and, at least, two of the following components: high blood pressure (BP), high triglycerides (TG), reduced HDL cholesterol (HDL-C), and elevated fasting plasma glucose (FPG) [5, 6]. Metabolic syndrome’s relevance to future health is its relationship with the development of Type 2 diabetes and cardiovascular diseases [6].To date, defining MetS’s prevalence in children has been challenging, given the different existing criteria described in the literature [6]. In a recent systematic review published by Sharma et al. [3], the prevalence of MetS in children and adolescents varied from 3.4% in normal-weight to 29% in the group with obesity.

Metabolic syndrome is associated with obesity. This situation is concerning because the number of children with obesity worldwide is expected to reach 250 million in 2030 [7]. The most widely used tools for detecting obesity and its cardiometabolic complications in children and adolescents is body mass index (BMI=Weight/Height^2^, kg/m^2^). However, the BMI presents a critical limitation; it is not able to differentiate between body fat mass (FM) and fat-free body mass (FFM) [8]. Traditionally, most of the research in the field of metabolic complications associated with obesity in children has focused on evaluating body fat because of its strong association with cardiometabolic risk [9–12]. It is important to note that children with obesity, defined by BMI, have shown not only an increased FM but also a higher FFM [13, 14].

FFM is also an essential component of body composition. It represents approximately 80% of the body weight, including bones and lean body mass (muscles, extracellular water, nerve tissue, and other cells that are not adipocytes or fat cells) [15]. Fat-free/lean body mass (LBM) is involved metabolically in active processes such as resting energy expenditure, glucose uptake, and myokine secretion, which improve insulin sensitivity and stimulate lipolysis [16]. Currently, how high levels of body fat are associated with increased insulin resistance, MetS, dyslipidemia, and Type 2 diabetes is clear; meanwhile, the effects of LBM in some outcomes from a metabolic point of view are unclear [17]. In 2016, a review by Perreault et al. [18] concluded that the evidence on the mechanisms that link FFM and glucose homeostasis is currently limited, probably because studies have been carried out mainly in adults when the metabolic complications have already been established. Therefore, the primary objective of this systematic review and meta-analysis was to examine the possible differences in FFM/LBM in children with and without IS/IR, or glucose tolerance (GT) or MetS.

## Methods

### Data sources and search strategy

This review was carried out following the guidelines for systematic reviews and meta-analyses (PRISMA) [19–21]. It was registered in the international database of prospectively registered systematic reviews (PROSPERO; http://www.crd.york.ac.uk/prospero) with the registration number CRD42019124734.

The search was carried out in the following databases: Medline/Pubmed (National Library of Medicine of the USA); Embase (Elsevier); Scopus (Elsevier); Web of Science [Core Collection / SciELO Science Citation Index] (Clarivate Analytics), and SciELO.org (FAPESP / CAPES / CNPq / Virtual Health Library / BIREME /Support Foundation to the Federal University of São Paulo-FapUnifesp).

The keywords used for the search (body composition, LBM, FFM, lean mass, lean tissue mass, lean body weight, skeletal muscle mass, muscle mass, fat-free mass index (FFMI), skeletal muscle mass index, muscle mass index, IS, IR, GT, metabolic syndrome x, MetS, infant, child, adolescent, and adolescence), were validated in MeSH (National of Library of Medicine of the USA controlled vocabulary thesaurus used for indexing articles for PubMed) and Emtree (controlled vocabulary thesaurus for biomedicine and life science for Embase). In addition, for these two databases (Medline/Pubmed and Embase), as well as for the others (Scopus, Web of Science [Core Collection and SciELO Citation Index], and SciELO.org), we used free terms or descriptors (keywords and phrases). These terms were searched under specific field codes in the title, abstracts, and keywords (depending on the search engine characteristics used) to retrieve most of the literature on the topic with terms not classified in the thesaurus. For our Medline/Pubmed and Embase searches, we added a highly sensitive filter to identify human studies.

For information retrieval, we applied an advanced search for each database using Boolean operators and wildcards, according to the characteristics and filters that each source provided for the queries. A search strategy was proposed that contemplated the grouping of related key terms through the “OR” operator and the crossings between the sets of words determined with the “AND” operators; within each set of terms, the corresponding wildcards were used; the asterisk character (*) as a truncation option and quotation marks (“), for a slightly more exact search of the phrases.

The search strategies were reviewed by another high-level information retrieval specialist prior to execution using the PRESS checklist [22], and are described in Tables S1 and S2.

### Inclusion criteria

We included studies that (1) evaluated children and adolescents aged 0 to 18, with and without IS, IR, GT, and MetS; (2) evaluated body composition, namely, LBM, FFM, LM, lean tissue mass, skeletal muscle mass, muscle mass, skeletal muscle mass index, muscle mass index and FFMI, and reporting the differences that included both with or without IS, IR, GT, and MetS; (3) evaluated the results of IS and/or IR, GT, and MetS, including HDL-C, blood pressure, glucose, waist circumference, triglycerides, and insulin; (4) have one of the following study designs: cross-sectional study, case-control, observational study, or randomized controlled trial study design; (5) were published in peer-reviewed journals; (6) conducted studies in humans; (7) conducted studies published in English or Spanish, and (8) conducted studies published up to 21 June, 2021.

Automatic alerts for each database were established to provide weekly updates of new literature until June 2021.

Reference lists of included articles were manually screened to identify additional studies.

### Exclusion criteria

We excluded (1) studies in children having diseases other than IS, IR, GT, and MetS; (2) studies without information regarding FFM/LBM and IS/IR/GT/MetS in children or adolescents, (3) studies in which FFM/LBM for the whole body or subtotal body were not available; (4) studies in adults and animals, and (5) those presented in languages other than Spanish or English.

### Search results

Once executed search strings, exported the information from each database was in bibliographic management formats (Pubmed format [Medline/Pubmed], RIS (Embase, SciELO), CSV (Scopus), and CIW (Web of Science [Core Collection / SciELO Citation Index]). The text files were saved in folders and consolidated through a desktop application used for text mining called VantagePoint - VP (Search Technology Inc. 2020); with VantagePoint - VP we removed the duplicate references from the debugging of diacritics, spaces, and special characters, then we removed in phases the duplicates (by title, by abstract, by DOI). The de-duplication method used is one of the many procedures used by information professionals, being systematic, rigorous, and reproducible Bramer [23]. The search returned a total of 7111 potentially eligible articles. Two reviewers (DC and II) independently examined each publication for possible inclusion based on title, abstract, and full text, according to the inclusion and exclusion criteria.

The discrepancies among the reviewers were resolved by consensus. The arbitration of a third reviewer was used for the unresolved discrepancies (AGB).

### Data extraction

Independently, two of the authors (DC and II) extracted data from each study, including the author, study date, study design, location, inclusion and exclusion criteria, participant data, methodology used to evaluate FFM/LBM and results. This information was recorded in a file developed with Microsoft Excel®, which was previously tested by the authors.

### Outcome assessment

As primary results, the means (M) and standard deviations (SD) of weight and/or height and/or FFM and/or LBM were registered for each group. If this information was not available in the original paper (*n* = 11, including [24–34], we contacted the corresponding authors to obtain the desired information. Four of them [26, 28, 29, 34] responded. In the studies by Gonzalez-Gil et al. and Rodríguez-Rodríguez et al. [24, 27], the mean values (M) and standard deviations (SD) were calculated from the first quartile, median, third quartile, and sample size values, following Wan’s [35] guidelines.

As secondary results, all those outcomes that were associated with FFM/LBM, such as lean-fat ration, irisin concentration, leptin, and LBMI-Z were registered.

### Quality assessment

The analysis of the studies’ quality was performed by two of the authors (DC and II) independently, using the following tools: 1) for cross-sectional studies, the BSA Medical Sociology Group quality evaluation tool [36], 2) for longitudinal studies, the scale created by Tooth et al. [37], 3) for clinical trials, the Cochrane Collaboration’s tool [38]. The results of the quality assessment are shown in Additional file 1: Tables S3, S4, and S5. Quality was rated as high, moderate, low, or very low according to the GRADE (Grading of Recommendations Assessment, Development and Evaluation) criteria [39]. Summary of findings table were constructed using GRADE pro GDT (GRADEpro Guideline Development Tool [Software], McMaster University, 2020 [developed by Evidence Prime Inc]) [40].

### Statistical analysis

For the data analysis, we used Review Manager 5.4.1 (The Nordic Cochrane Centre, The Cochrane Collaboration, Copenhagen, Denmark) Software to calculate the standardized mean difference (SMD) with 95% confidence intervals (CI). The standardized mean difference for continuous data (FFM/LBM (kg) or (%)) in each study was calculated and combined using the random-effects model (DerSimonian and Laird approach). In the studies in which there was a double comparison, obese vs. obese and normal weight vs. obese, both comparisons were included in the meta-analysis.

Positive effect sizes indicated higher FFM/LBM (kg) or (%) in individuals with IS/IR/GT/MetS compared to individuals without IS/IR/GT/MetS. Negative effect sizes indicated lower FFM/LBM (kg) or (%) in individuals with IS/IR/GT/MetS compared to individuals without IS/IR/GT/MetS.

Heterogeneity between trial results was tested using the heterogeneity index (I^2^) whose thresholds for interpretation are < 25%, low heterogeneity; 50 to 75% may represent moderate heterogeneity; > 75% may represent high heterogeneity [41]. The *p*-value associated with the studies’ heterogeneity was calculated, indicating a non-significant result as the absence of heterogeneity. Leave-one-out sensitivity analyses were performed to assess the influence of outliers in FFM/LBM (%) and FFM/LBM (kg) using Open Meta [Analyst] software.

Publication bias was assessed by Egger’s test following the indications provided by Peters et al. [42]. Additionally, the Begg and Mazumdar test was applied to measure asymmetry in funnel plots [43].

## Results

**Fig. 1:**
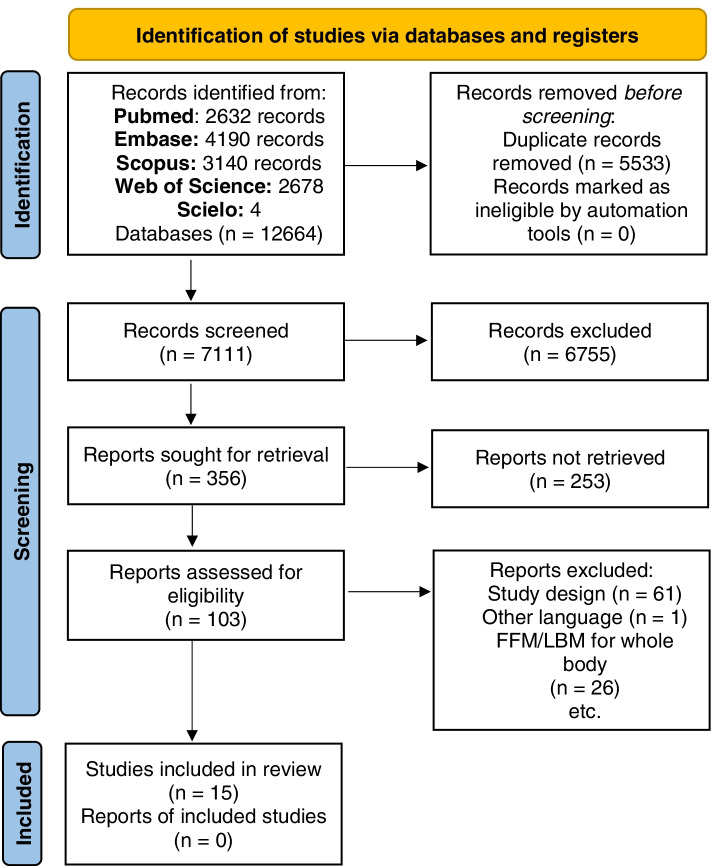
PRISMA 2020 flow diagram for new systematic reviews which included searches of databases and registers only. *From:* Page MJ, McKenzie JE, Bossuyt PM, Boutron I, Hoffmann TC, Mulrow CD, et al. The PRISMA 2020 statement: an updated guideline for reporting systematic reviews. BMJ 2021;372:n71. doi: 10.1136/bmj.n71. For more information, visit: http://www.prisma-statement.org/

Finally, the following 103 articles were selected: [24–34, 44–135] after the evaluation of the full texts, 78 were excluded for the following reasons: (1) the design of the studies did not meet the inclusion criteria defined for this review [44–46, 48–53, 55–57, 59–61, 65, 66, 68–73, 79, 81, 84, 86, 88–91, 97, 100–104, 106, 108, 110, 112–117, 119–122, 124–132, 134, 135] (2) the studies did not present an evaluation of whole body FFM/LBM, these were [47, 54, 58, 62–64, 67, 74–78, 80, 82, 83, 85, 92–96, 105, 107, 109, 111, 123] and (3) another language [87] (Fig. 1).

### Characteristics of the included studies

This review includes the results of 15 studies. Eleven of them [24, 25, 27, 29, 30, 32–34, 98, 99, 118] were cross-sectional studies (CS). One [31] was a longitudinal study, and three [26, 28, 133] were clinical trials (CT) (Fig. 1), which included a total of 5642 children (51.8% boys). The information included in this systematic review and meta-analysis corresponds to the baseline data for the longitudinal studies and clinical trials to make them suitable to compare or combine in this systematic review.

Regarding the quality assessment, ten (90.9%) of the cross-sectional studies [24, 25, 27, 29, 32–34, 98, 99, 118] received a moderate overall rating. Weiss et al. [30] had a high overall rating (9.1%), see Additional file 1: Table S3. According to the scale by Tooth et al. [37], the longitudinal study [31] had a low score (13/33), see Additional file 1: Table S4. According to the Cochrane Collaboration’s tool [38] for assessing risk, the three clinical trials [26, 28, 133] presented bias risk. However, it is unlikely for this review that it affected the results because the included information corresponds to the baseline data before the intervention took place (Additional file 1: Table S5). Quality of evidence across studies was evaluated for each outcome using the GRADE approach [39]. A summary of findings table is presented in Additional file 1: Table S6.

### Participants sample size, country, and age

The sample sizes of the studies included in this review ranged from *n* = 28 to *n* = 3004 participants [30, 34]. Regarding the countries where the studies were developed, five [25, 30, 33, 34, 98] were carried out in the USA, two [31, 32] in Italy, and two [26, 28] in Brazil. One study was performed in Mexico [24], Spain [27]; Chile [29]; France [133]; Iran [99]; and Turkey [118].

The age of the participants ranged from 5.2 to 19 years.

### Maturation stage

Ten studies [25, 26, 28, 30–33, 98, 99, 133] provided information on the methodology used for the assessment of the maturation stage; the Tanner scale was the method most widely used. Of the population referred to in these studies, 28.7% (*n* = 349) were in Tanner I stage, that is, [30–33, 98, 99] and 73.3% (*n* = 959), in Tanner stages from II to V, namely, [25, 26, 28, 30, 32, 33, 98, 99, 133].

### FFM/LBM measurement techniques

There are several body composition techniques available for the estimation of FFM/LBM in infants, children, and adolescents, including anthropometric measurements, bioelectrical impedance analysis (BIA), air-displacement plethysmography (ADP), dual-energy X-ray absorptiometry (DXA), computerized tomography (CT), magnetic resonance imaging (MRI), and ultrasound techniques [136]. In this review, one (*n* = 443) study [27] used anthropometric measurements. Three (*n* = 280) studies [24, 99, 118] used BIA. Eight studies (*n* = 4640) [25, 29–32, 34, 98, 133] used DXA, and three (*n* = 279) studies, [26, 28, 33] used air displacement plethysmography (BOD-POD).

Seven studies [28–30, 32–34, 98] registered LM, LBM, or lean tissue mass, which refers to the fat-free and bone mineral-free component, including muscles, skin, tendons, and connective tissues [137]. Seven studies [24–27, 31, 118, 133] expressed the results in terms of FFM, defined as the sum of muscle mass, bones, internal organ non-adipose components, and extracellular fluid [138]. One study expressed the results in terms of muscle mass [99].

Regarding the used indices to assess FFM/LBM, from the 14 studies included in the review, three [28, 29, 33] described LBM or lean tissue mass (%). Three studies [30, 33, 98] described LBM or lean tissue mass (kg). one study [34] described LBMI-Z, and another [32] described LBMI (kg/m^2^).

Regarding FFM, four studies [26, 27, 31, 118] described FFM (%). Six studies [24–26, 31, 118, 133] described FFM (kg) and, one study [118], FFMI.

Gonzalez-Gil et al. study [24] described lean-fat ratio calculated as the quotient of muscle mass (kg) and fat mass (kg) and [24, 99] muscle mass (kg).

The results of the individual studies are presented in Tables 1, 2 and 3.

**Table 1:** Studies investigating the association between FFM/LBM and IR in children and adolescents

Reference	Study design	Population n (♀;♂)	Age	Country	Study period	Method to assess maturation stage	Method to assess body composition	Metabolic variables	IR criteria	Body composition outcome by IR		Association: body composition– IR	Secondary outcomes
										Non IR mean ± SD	IR mean ± SD		
Burrows et al. [30]	CS	667 adolescents (♀ 47.8%; ♂ 52.2%)	16.8 ± 0.3	Chile	NA	NA	DXA	Fasting glucose; TG; HDL-C; WC; BP; adiponectin; hs-CRP.	HOMA-IR	(*n* = 558)	(*n* = 109)	Adolescents with IR had significantly lower (*p* < 0.001) mean values of LM (%).	Independently significant association between IR and sarcopenia (OR: 4.9; 95% CI: 3.2–7.5)
										LM (%) 68.7 ± 11.4	LM (%) 62.0 ± 9.4		
Sanches et al. [29]	CT	66 post-pubertal adolescents with obesity.	16.8 ± 1.6	Brazil	Tanner V 100%	Tanner stages	Air-displacement plethysmography (BOD-POD)	LDL-C; HDL-C; VLDL; HOMA-IR; QUICKI; MBP; leptin; adiponectin; Leptin/Adiponectin ratio and resistin.	HOMA-IR; QUICKI	(*n* = 27)	(*n* = 39)	No significant difference between groups with IR and non-IR.	
										LBM (%) 52.62 ± 5.77	LBM (%) 52.42 ± 5.34		
Rodríguez-Rodríguez et al. [28]	CS	443 schoolchildren (♀ 44.4%; ♂ 55.5%)	10 (9–11)	Spain	NA	NA	Anthropometric measurements (equation of Parizkova for body fat (%))	Fasting glucose; TG; HDL-C; WC; BP; adiponectin determinations; hs-CRP.	HOMA	(*n* = 427) (♀ 238; ♂ 189)	(*n* = 16) (♀ 8; ♂ 8)	Adolescents with IR had significantly lower (*p* < 0.01) mean values of FFBM (%) and significantly differences with respect to sex (*p* < 0.05)	
										FFBM (%) ♀ 77.8 (72.1–82.7) ♂ 79.8 (74.2–85.4) Total 78.5 (73.0–83.8)	FFBM (%) ♀69.5 (67.4–74.0) ♂ 72.9 (69.6–81.9) Total 70.6 (68.2–75.8)		

*CS* Cross-sectional studies, *CT* Clinical trial, *NA* Not available, *IR* Insulin resistance, *TG* Triglycerides, *HDL-C* High density lipoprotein cholesterol, *WC* Waist circumference, *BP* Blood pressure, *hs-CRP* High sensitivity C-reactive protein, *LDL-C* Low density lipoprotein cholesterol, *VLDL* Very low-density lipoprotein, *HOMA-IR* Homeostasis model assessment insulin resistance index, *QUICKI* Quantitative insulin sensitivity check index, *MBP* Mean blood pressure, *GF/IF* The ratio of fasting glucose to fasting insulin, *DXA* Dual energy X-ray absorptiometry, *LM* Lean mass, *LBM* Lean body mass, *TLM* Total lean mass, *FFBM* Fat free body mass

**Table 2:** Studies investigating the association between FFM/LBM and GT in children and adolescents

Reference	Study design	Population n (♀; ♂)	Age	Country	Study period	Method to assess maturation stage	Method to assess body composition	Metabolic variables	GT criteria	Body composition outcome by GT.		Association: body composition– GT	Secondary outcomes
										NGT Mean ± SD	IGT Mean ± SD		
Kim, et al. [25]	CS	205 adolescents (♀ 66%; ♂ 34%)	14.6 ± 0.2	USA	NA	Tanner scale IV 30% (*n* = 61); V 70% (*n* = 115)	DXA	Glucose; HOMA-IR; insulin; free fatty acids; HbA1c; lipid profile; leptin and adiponectin.	HbA1c and/or a 2-h oral glucose tolerance test (OGTT)	(*n* = 138)	(*n* = 38)	FFM was progressively and significantly higher from normal weight to obese and from NGT to IGT (*p* < 0.0001)	
										Normal weight (*n* = 49) Tanner IV (55% *n* = 27); V (45% (*n* = 22) FFM (kg) 41.3 ± 1.2	Obese (*n* = 38) Tanner IV (21% *n* = 8); V (79% *n* = 30) FFM (kg) 51.4 ± 1.7		
										Obese (*n* = 89) Tanner IV (29% *n* = 26); V (71% *n* = 63) FFM (kg) 49.2 ± 1.0			
Goran et al. [99]	CS	150 children and adolescents (♀ 43.3% ♂ 56.7%)	11.0 ± 1.7	USA	NA	Tanner stages I (36% *n* = 54) II (33.3% n = %50) III (8.6% *n* = 13) IV (12% *n* = 18) V (9.3% *n* = 14)	DXA	Fasting glucose; 2-h oral glucose tolerance test; fasting insulin	2-h oral glucose tolerance test (OGTT)	(*n* = 87)	(*n* = 35)	No significant difference between groups with NGT and IGT	
										LBM (kg) 35.9 ± 10.3	LBM (kg) 36.3 ± 10.0		
Weiss et al. [31]	CS	28 children and adolescents with obesity (♀ 57.1; ♂ 42.8)	13.5 ± 2.1	USA	NA	Tanner stages. PrepubertaTanner (28.6% *n* = 8); pubertal (71.4% *n* = 20).	DXA	Fasting glucose; 2 h glucose; fasting insulin; fasting C-peptide; leptin; adiponectin; HbA1c; plasma fatty acids; glycerol; glycerol turnover, and lipid oxidation rates.	Euglycaemic hyperinsulinaemic clamp and the hyperglycaemic clamp.	(*n* = 14) (♀ 6; ♂ 8)	(*n* = 14) (♀ 6; ♂ 8)	No significant difference between groups with IGT and NGT	
										LBM (kg) 55.9 ± 9.4	LBM (kg) 53.2 ± 15.2		

*CS* Cross-sectional studies, *NGT* Normal glucose tolerance, *IGT* Impaired glucose tolerance, *DXA* Dual energy X-ray absorptiometry, *LBM* Lean body mass, *HOMA-IR* Homeostasis model assessment insulin resistance index, *HbA1c* Glycated hemoglobin, *OGTT* Oral glucose tolerance test, *FFM* Fat-free mass

**Table 3:** Studies investigating the association between FFM/LBM and MetS in children and adolescents

Reference	Study design	Population n (♀; ♂)	Age	Country	Study period	Method to assess maturation stage	Method to assess body composition	Metabolic variables	MetS criteria	Body composition outcome by MetS		Association: body composition– MetS	Secondary outcomes
										No MetS mean ± SD	MetS mean ± SD		
Khammassi et [42].	CT	92 adolescents with obesity	12-25	France	NA	Tanner stages 3-4	DXA	Glucose, insulin, TG, TC, HDL-c, LDL-c, HOMA-IR, WC and BP.	Based by Chen et al.	(*n* = 44) FFM (kg) 48.52 ± 7.24	(*n* = 40) FFM (kg) 55.49 ± 7.34	FFM was significantly higher in the MetS group *p* < 0.001.	
Behrooz et al. [100]	CS	90 children and adolescents (♀ 51.1% ♂;48.9%)	10-18	Iran	2019	Tanner stages.	BIA	fasting glucose, insulin, lipid profile, spexin, high-sensitivity C-reactive protein (hs-CRP) and HOMA-IR	Based by Cook et al.	(*n* = 76)	(*n* = 14)	No significant difference between groups with MetS and non-MetS.	
										Muscle mass(kg) 40.45 ± 15.06	Muscle mass(kg) 52.90 ± 13.52		
Gonzalez-Gil et al. [24]	CS	46 normal weight, 40 obese, and 40 MetS children (♀ 51.6%; ♂ 48.4%)	6-12	Mexico	NA	NA	BIA and anthropometric measurements	BP, irisin, leptin, adiponectin, adipsin, resistin, TG, fasting glucose, HDL-c) levels, and WC.	Based by Cook et al.	(*n* = 86)	(*n* = 40)	Muscle mass, FFM was significantly higher in the obese and MetS groups compared to control group (normal weight)	Lean-fat ration (muscle mass (kg)/fat mass (kg)) was significantly lower in the obese 0.433 (0.380–0.627) and the MetS group 0.447 (0.345–.610) compared with the normal weight group 1.68 (1.25–2.01) Negative correlations between plasma irisin concentration and FFM (rs = − 0.257) were found. The noteworthy, lean-fat ratio was found to have a positive correlation with irisin (0.489; *p* < 0.001). Leptin was found to be positively correlated with, FFM (rs = 0.329) and negative correlation with lean-fat ratio (rs = − 0.376).
										Normal weight (*n* = 46) FFM (kg) 23.05 (20.3–26.8) Muscle mass (kg) 6.43 (5.4–7.9)	Obese (*n* = 40) FFM (kg) 29.06 (24.4–32.8) Muscle mass (kg) 7.58 (6.5–9.0)		
										Obese (*n* = 40) FFM (kg) 27.22 (23.9–31.4) Muscle mass (kg) 6.56 (5.8–7.5)			
Masquio et al. [27]	CT	108 postpuberty obese adolescents	15-19	Brazil	2004	Tanner scale. Postpuberty Tanner ≥V 100%	Air- Displacement plethysmography BOD-POD	Glucose, insulin, TG, TC, HDL-c, LDL-c, leptin, adiponectin, PAI-1, CRP, ICAM-1, VCAM-1, (L/A ratio), (A/L ratio), HOMA-IR, QUICKI, WC, BP.	International Diabetes Federation criteria	(*n* = 76)	(*n* = 32)	MetS group presented significantly higher FFM (kg) *p* < 0.05.	
										FFM (%) 54.87 ± 7.02	FFM (%) 54.60 ± 6.31		
										FFM (kg) 54.62 ± 9.48	FFM (kg) 59.97 ± 8.28		
Weber et al. [26]	CS	3004 (♀ 44%; ♂ 56%)	16.1 ± 2.5	USA	1999-2006	NA	DXA	Fasting glucose; insulin; TG; HDL-C; WC; BP.	International Diabetes Federation criteria	(*n* = 2835)	(*n* = 169 ♀ 5.1%; ♂ 6.8%)	Participants with MetS had significantly greater LBMI compared with participants No MetS (*p* < 0.0001).	LBMI-Z was significantly associated with a greater odds of low HDL-C(1.5; 95% CI 1.2–1.9), elevated BP (1.8; 95% CI: 1.1–2.9), high WC (3.7; 95% CI: 2.4 –5.9), and elevated fasting insulin (1.8; 95% CI 1.4 –2.5), independent of FMI-Z.
										LBMI-Z −0.07 ± 0.96	LBMI-Z 1.09 ± 0.92		
Ayvaz et al. [119]	CS	32 normal weight and 32 children with obesity (♀ 35.9%; ♂ 64.0%)	13.6 ± 2.1	Turkey	2007	NA	BIA	Fasting glucose; TG; HDL-C; WC; BP; creatinine; uric acid; total protein; albumin; SGOT; SGPT; serum lipids; C-reactive protein; fibrinogen; fasting insulin level; TSH and HOMA-IR.	Ianuzzi	(*n* = 17 obese children)	(*n* = 15 obese children)	Obese children with MetS had significantly lower (*p* < 0.05) mean values of FFM index. No significant difference of FFM and FFM% between the groups with MetS and No MetS.	
										FFM (kg) 42.65 ± 9.38	FFM (kg) 49.24 ± 13.17		
										FFM (%) 0.69 ± 0.05	FFM (%) 0.67 ± 0.07		
										FFMI (kg/m^2)^ 19.63 ± 2.18	FFMI (kg/m^2)^ 18.14 ± 1.82		
Brufani et al. [33]	CS	439 children and adolescents with obesity (♀ 51.5%; ♂ 48.5%)	5.2–17.9	Italy	2003-2010	Tanner stages. PrepubertaTanner stage I (45.8% *n* = 201); pubertal Tanner stage II-V (54.2% *n* = 238).	DXA	Glucose; insulin; C peptide; HDL-C; TC; TG; ISI; OGTT; DI; BP.	Based on the National Cholesterol Education Program	(*n* = 177)	(*n* = 24)	No significant difference of LBMI between the groups with MetS and No MetS.	LBMI to be positively associated with MetS (*p* = 0.004)
										Prepubertal LBMI (kg/m^2^) 15.2 ± 1.5	Prepubertal LBMI (kg/m^2^) 15.7 ± 1.5		
										(*n* = 187)	(*n* = 51)		
										Pubertal LBMI (kg/m^2^) 17.6 ± 2.4	Pubertal LBMI (kg/m^2^) 18.2 ± 2.7		
Hsu et al. [34]	CS	105 (♀ 75%; ♂ 25%)	13 ± 3	USA	2009	Tanner stages I (18.1% *n* = 19) II (19.0% *n* = 20) III (3.8% *n* = 4) IV (17.1% *n* = 18) V (41.9% *n* = 44)	Air- Displacement plethysmography BOD-POD	Fasting glucose; HDL-C; TG; BP and WC.	Based on Cruz et al. and Cook et al.	(*n* = 88) Tanner I (21.6% *n* = 19); II (19.3% *n* = 17); III (2.3% *n* = 2); IV (13.6% *n* = 12); V (43.2% *n* = 38)	(*n* = 17) Tanner I (0% *n* = 0); II (17.6% *n* = 3); III (11.8% *n* = 2); IV (35.3% *n* = 8); V (35.3% *n* = 8)	Participants with MetS had greater total lean tissue mass (*p* = 0.02) and lower percent lean tissue mass (*p* = 0.002)	
										Total lean tissue mass (kg) 45.96 ± 16.25	Total lean tissue mass (kg) 56.03 ± 14.02		
										Lean tissue mass (%) 67.79 ± 10.97	Lean tissue mass (%) 58.79 ± 8.71		
Brufani et al. [32]	LS	55 prepubertal children with obesity (♀ 36,3%; ♂ 63.6%)	9.6 ± 1.3	Italy	2004-2006	Marshall and Tanner I (100% *n* = 55)	DXA	Fasting glucose; insulin; TG; HDL-C; BP; HOMA-IR; QUICKI; ISI	Weiss et al.	(*n* = 37) Tanner I (100% *n* = 37)	(*n* = 8) Tanner I (100% *n* = 8)	No significant difference between groups with MetS and No MetS.	
										FFM (%) 56.3 ± 3.1	FFM (%) 55.8 ± 4.3		
										FFM (kg) 31.4 ± 6.5	FFM (kg) 30.8 ± 5.0		

*CS* Cross-sectional studies, *LS* Longitudinal study, *NA* Not available, *MetS* Metabolic syndrome, *No MetS* No metabolic syndrome, *TG* Triglycerides, *HDL-C* High density lipoprotein cholesterol, *TC* Total cholesterol, *WC* Waist circumference, *BP* Blood pressure, *SGOT* Serum glutamic oxaloacetic transaminase, *SGPT* Serum glutamic pyruvic transaminase, *TSH* Thyroid-stimulating hormone, *ISI* Insulin sensitivity index, *OGTT* Oral glucose tolerance test, *DI* Disposition index, *HOMA-IR* Homeostasis model assessment insulin resistance index, *QUICKI* Quantitative insulin sensitivity check index, *DXA* Dual energy X-ray absorptiometry, *BIA* Bioelectrical impedance analysis, *LBMI* Lean body mass index, *FFMI* Fat free mass index, *FFM* Fat-free mass

### Methodologies of glucose homeostasis measurement

For glucose homeostasis, several tests exist to assess the in vivo action of insulin, involving model evaluations, glucose sensitivity studies, and insulin and glucose clamps. These are fasting plasma glucose (aFPG), fasting plasma insulin resistance (FPI), insulin resistance (IR), homeostatic model assessment (HOMA), quantitative insulin sensitivity check index (QUICKI), meal tolerance test (MTT), oral glucose tolerance test (OGTT), intraperitoneal insulin sensitivity test (IPIST), and intraperitoneal glucose tolerance test (IPGTT) [139]. However, the hyperinsulinemic-euglycemic clamp is the gold standard [140].

In this review, three (*n* = 366) studies [24, 98, 99] examined glucose homeostasis using fasting plasma glucose. Two studies (*n* = 355) [25, 98] used an oral glucose tolerance test (OGTT). Four (*n* = 3603) studies used other indices, such as the ratio of fasting glucose to fasting insulin (GF/IF); these were [31–34]. Eight (*n* = 1140) studies [26–29, 31, 99, 118, 133] used the homeostasis model assessment insulin resistance index (HOMA-IR). And, the quantitative insulin sensitivity check index (QUICKI) was used in three (*n* = 229) studies [26, 28, 31]. See Tables 1, 2 and 3.

A study by Weiss et al. [30] used the euglycaemic hyperinsulinaemic and hyperglycaemic clamps, in which case, the term glucose tolerance was used (n = 28). See Table 2.

To summarize, three studies [27–29] investigated FFM/LBM in children and adolescents using IR. Three [25, 30, 98] involved children and adolescents with GT problems, and nine [24, 26, 31–34, 99, 118, 133] involved children and adolescents with MetS.

### FFM/LBM differences according to IR, GT, or MetS

**Fig. 2:**
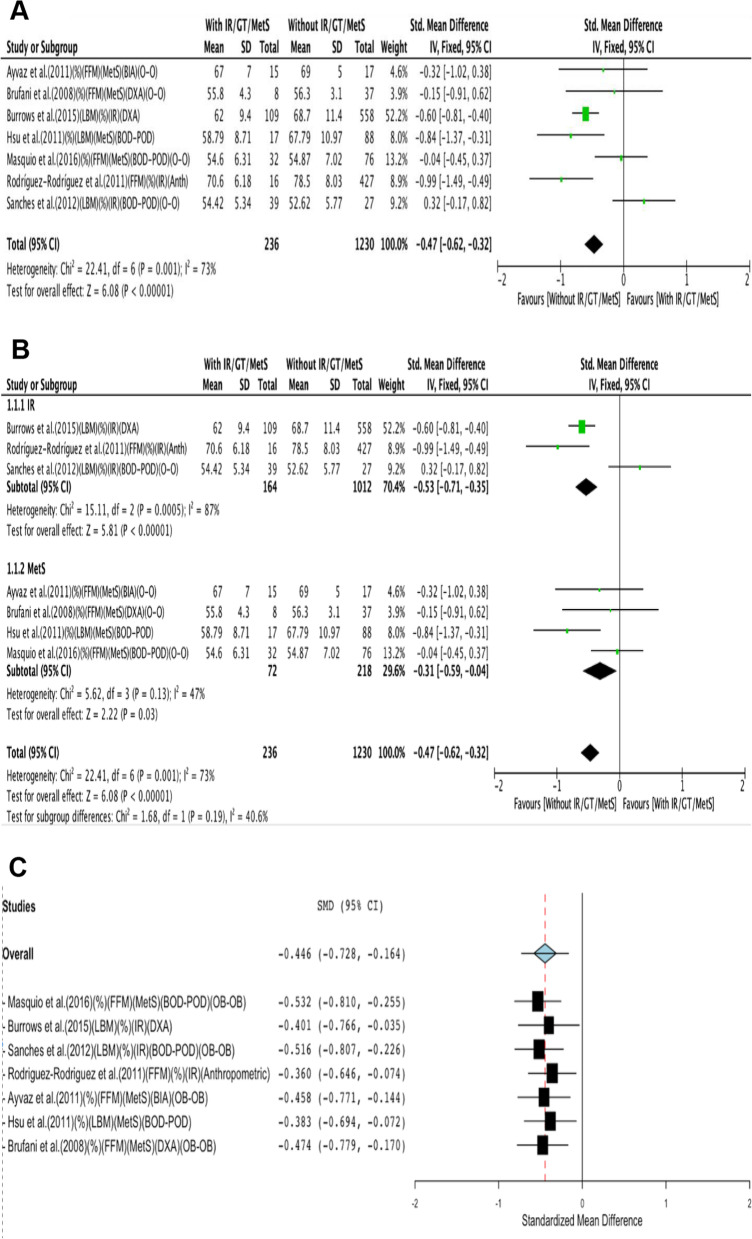
Random-effects meta-analysis with IR/MetS or without IR/MetS on FFM/LBM (%). **a** FFM/LBM (%) **b** Subgroup analyses by diagnosis (group IR and group MetS). **c** Leave-one-out meta-analysis. Abbreviations: FFM, fat-free mass; LBM, lean body mass; IR, insulin resistance; MetS, metabolic syndrome

When performing the meta-analysis, using the three metabolic conditions together, it was observed that individuals with IR/GT/MetS had lower FFM/LBM (%) than those without IR/GT/MetS (SMD -0.47; 95% CI, −0.62 to −0.32; Fig. 2A). The heterogeneity between the studies was moderate (I^2^ = 73; *p* = 0.001), According to the GRADE system, the certainty of the evidence was very low.

**Fig. 3:**
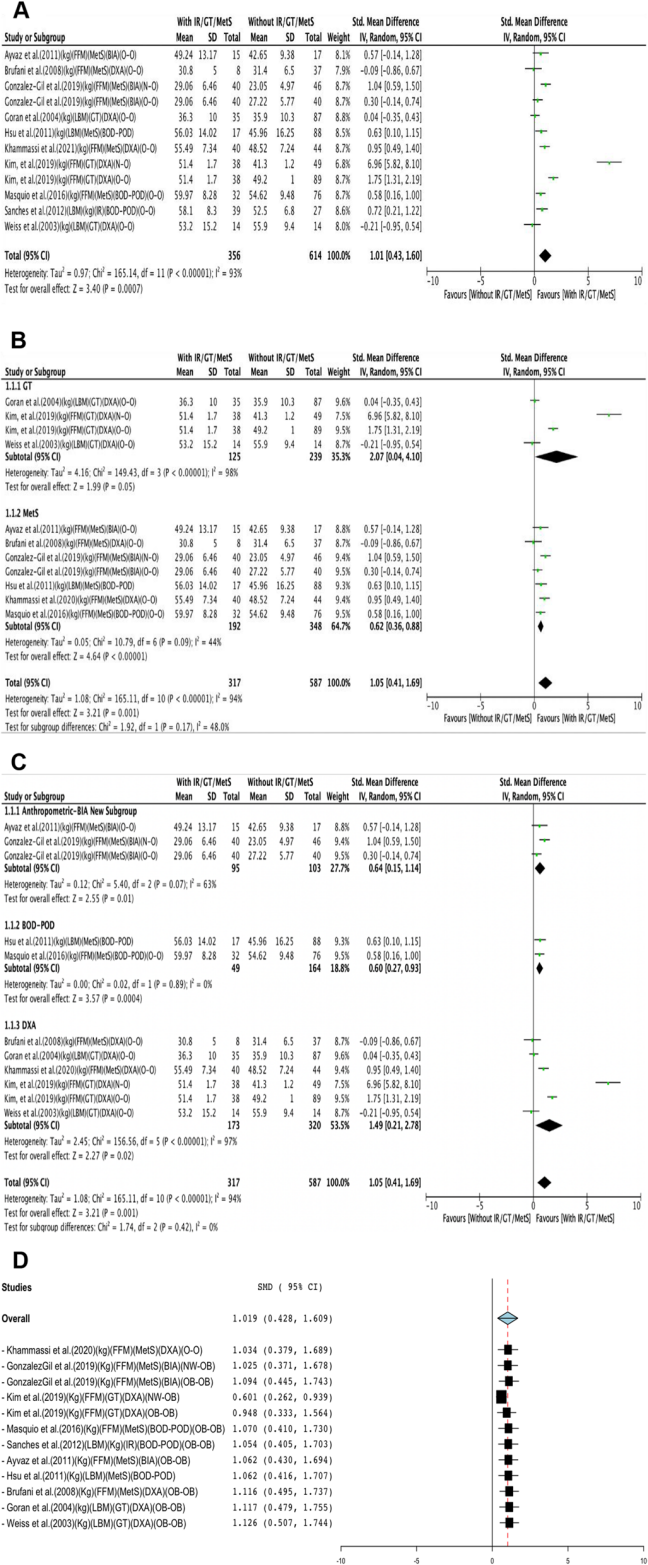
Random-effects meta-analysis with IR/GT/MetS or without IR/GT/MetS on FFM/LBM (kg). **a** FFM/LBM (kg) **b** Subgroup analyses by diagnosis (group GT and group MetS). **c** Subgroup analysis by a device (anthropometric measurements and BIA group and BOD-POD and DXA group). **d** Leave-one-out meta-analysis. Abbreviations: FFM, fat-free mass; LBM, lean body mass; IR, insulin resistance; GT, glucose tolerance; MetS, metabolic syndrome

Figure 3A shows the analysis of FFM/LBM (kg) absolute values in the participants with or without IR/GT/MetS. The group with IR/GT/MetS had a higher FFM/LBM (kg) (SMD, 1.01; 95% CI, 0.43 to 1.60) compared to the group without IR/GT/MetS. The heterogeneity was high (I^2^ = 93; *p* = < 0.001).

A sensitivity analysis was performed including only one comparison per study (obese vs. obese; excluding the obese vs. normal-weight comparison), the results were consistent; the group with IR/GT/MetS still had a higher FFM/LBM (kg) (MSD, 0.55; 95% CI, 0.19 to 0.92) when compared to the group without IR/GT/MetS. High heterogeneity was found (I^2^ = 81, *p* = < 0.001). The quality of evidence for this outcome was low.

### FFM/LBM and insulin resistance

In the meta-analysis (Fig. 2B), subgroup analysis results suggested a lower FFM/LBM (%) in the group of participants with IR (SMD, −0.53; 95% CI, −0.71 to −0.35) with high heterogeneity between studies (I^2^ = 87; *p* < 0.01). According to the GRADE system, the certainty of the evidence was very low.

Because each of the three included studies for the IR group used a different measurement technique. A subgroup analysis taking into account the used body composition device could not be performed.

Only the study by Sanches et al. [28] included data for FFM/LBM (kg). Therefore, we were not able to perform a meta-analysis for this variable in IR children.

### FFM/LBM and GT

We were not able to estimate the differences in the FFM/LBM (%) in individuals with or without GT because no studies reported FFM/LBM (%) in this group. However, for FFM/LBM (kg), Fig. 3B shows that there were statistical significant differences in FFM/LBM (kg) between both groups (SMD, 2.07; 95% CI, 0.04 to 4.10), with high heterogeneity (I^2^ = 98; p < 0.01). The results were not consistent when performing the analysis that included a comparison per study (obese vs. obese; excluding the obese vs. normal-weight comparison); there were no statistically significant differences between both groups (SMD, 0.54; 95% CI, −0.72 to 1.81), and high heterogeneity (I^2^ = 95; *p* < 0.01). The quality of evidence for this outcome was very low.

### FFM/LBM and MetS

In the meta-analysis (Fig. 2B), the results of the subgroup analysis showed that there were statistical significant differences in FFM/LBM (%) between both groups (SMD, −0.31; 95% CI, −0.59 to −0.04), with low heterogeneity (I^2^ = 47; *p* = 0.13) and the quality of evidence was very low.

Figure 3B shows the subgroup analysis for FFM/LBM (kg) in the participants with or without MetS, suggesting higher values of FFM/LBM (kg) in the group of participants with MetS (SMD, 0.62; 95% CI, 0.36 to 0.88) with low heterogeneity (I^2^ = 44; *p* = 0.09). The quality of evidence for this outcome was low.

Regarding the analysis by subgroups, taking into account the type of device used to assess the FFM/LBM (kg), significantly higher FFM/LBM (kg) values were found in the groups with MetS when evaluated with anthropometry-BIA (SMD, 0.64; 95% CI, 0.15 to 1.14; Fig. 3C) with moderate heterogeneity (I^2^ = 63; *p* = 0.07), BOD- POD (SMD, 0.60; 95% CI, 0.271 to 0.93; Fig. 3C) with low heterogeneity (I^2^ = 0.0; *p* = 0.89), and DXA (SMD, 1.49; 95% CI, 0.21 to 2.78; Fig. 3C) with high heterogeneity (I^2^ = 97; *p* < 0.01) and the quality of evidence was very low.

The results were consistent when performing the analysis including a comparison per study (obesity vs. obesity, excluding the obese vs. normal-weight comparison); higher values of FFM/LBM (kg) were found in the group of participants with MetS (SMD, 0.44; 95% CI, 0.21 to 0.68) with low heterogeneity (I^2^ = 0; *p* = 0.51) and the quality of evidence was low.

When performing the analysis, including a comparison per study (obesity vs. obesity, excluding the obese vs. normal-weight comparison), no significant differences were found between groups when measured using anthropometry-BIA and DXA.

### Sensitivity analysis

A forest plot was conducted leaving one out of the total included studies for each combination as a sensitivity analysis. The effect size remained significant after the omission of each study from the meta-analysis (Figs. 2C and 3D).

### Publication bias

No evidence of publication bias was found for studies measuring FFM/LBM (%) (Begg’s *p* = 1.000; Egger’s *p* = 0.681). For the studies that included measurements of FFM/LBM (kg), no evidence of publication bias was found according to the Begg test (*p* = 1.000), but we have obtained significant results of possible bias when the Egger test was used (*p* = 0.006), although the reduced number of included studies could limit this analysis.

To summarize, the meta-analysis showed lower FFM/LBM values (%) in participants with IR/GT/MetS. This situation can also be seen in the diagnostic subgroup analyses in the case of IR and MetS. Regarding FFM/LBM behavior, when expressed in (kg), the meta-analyses showed higher values in the group with IR/GT/MetS; this could also be evidenced in the subgroup analyses by diagnosis in the case of GT and MetS. Regarding the device used for its measurement, significant differences were found between groups with MetS when evaluated with anthropometry-BIA, BOD-POD and DXA. For IR, it was impossible to show the difference because the analysis by subgroups could not be performed, given the low number of studies included in this systematic review.

## Discussion

To the best of our knowledge, this is the first systematic review and meta-analysis providing an overview of current scientific evidence regarding the possible differences between FFM/LBM in children with and without IR, glucose tolerance, or MetS.

Our systematic review and meta-analysis identified lower values of FFM/LBM (%) in children and adolescents with IR/GT/MetS, and higher values of FFM/LBM when these are expressed in kg. Considering that the percentage of LBM/FFM automatically decreases in proportion to increases in % of body fat [141], it is likely that children and adolescents with IR/GT/MetS will present higher values of body fat and android fat (visceral fat) accumulation, as shown in adults [142]. This will eventually lead to lower values of FFM/LBM in proportion to the total body weight. This effect on glucose homeostasis could be entirely or largely due to the association between adiposity and insulin resistance, previously described in children [143].

In this review, two studies [27, 29] found that adolescents with IR had significantly lower FFM/LBM (%). This could be due to the fact that FFM is a metabolically active tissue associated with insulin-stimulated glucose uptake in the postprandial state in humans, as well as greater insulin sensitivity [144], reduction in the accumulation of fat inside the muscle [145], and muscle secretory products or “myokines” that favor IS [16].

Furthermore, low muscle mass has been associated with cardiovascular risk factors, such as increased blood pressure, risk of abdominal obesity, and hypertriglyceridemia [122], as well as arterial stiffness [146] and low muscle fitness, which, in turn, has been independently associated with metabolic risk in children and adolescents [147].

Finally, this systematic review identified six studies that found higher levels of FFM/LBM/muscle mass in children with MetS or GT. Five (*n* = 3435) of them [24, 26, 33, 34, 133] found higher levels in children with MetS, and one (*n* = 205) with glucose tolerance [25]. Other available studies have described higher levels of FFM/LBM in individuals with MetS. You et al. [148] found that in postmenopausal women, 50 to 70 years old, lean mass (kg) was significantly higher (*p* < 0.05) in the group of women with higher HOMA-IR scores and MetS compared to those without MetS (44.4 ± 0.9; 41.2 ± 0.9). Brochu et al. [142], in a study with 43 postmenopausal and sedentary women, found that women with metabolically abnormal obesity MAO (low IS) showed higher levels of LBM (kg) than those who were metabolically healthy but obese (MHO) (43.8 ± 5.5; 48.1 ± 7.2 [*p* < 0.03]). These findings are in line with our meta-analysis findings. People presenting these diseases generally have a higher weight and, consequently, higher absolute lean mass values. Nonetheless, as shown in this meta-analysis, relative values (%) are generally similar or even lower, which, as stated above, represent not only the values on FFM/LBM but also the proportion of fat mass.

On the other hand, other studies have shown that increased muscle mass does not necessarily translate into better muscle quality or better physical performance in people with MetS. This is the case of the study developed by Mesinovic et al. [149] with overweight and obese older adults. They observed that people with MetS had lower muscle quality (muscle density and strength normalized to lean mass) despite having a higher FFM. The previous suggests that a higher FFM does not confer an advantage from a functional point of view. Similarly, the study carried out with 1050 adolescents participating in the Korean National Health and Nutrition Examination Survey found a lower handgrip-to-weight ratio in adolescents with metabolic syndrome [67].

The mechanisms underlying the association between FFM/LBM and IR, GT or MetS are not entirely clear. In the case of adults, it is attributed to the types of fibers (higher percentage of type II and type IIx muscle fibers) whose capillary density is reduced, which limits the transport of glucose to the muscles; as well as a reduced oxidative capacity and an increase in intramuscular fat storage [150]. However, more children-focused studies are needed to examine these mechanisms in the early stages of life.

### Limitations and strengths

This systematic review and meta-analysis presents some limitations. The first one involves the different terms found in the literature to define both FFM and LBM [151] and IR, GT, and MetS [152], especially in pediatric populations.

The second is related to the investigations’ population heterogeneity. Some of the studies focused on patients with obesity, while others included children and adolescents with both normal BMI and obesity, in different age ranges, maturation stages, grouping them by gender, or combining the two. Some presented heterogeneous methodologies for determining body composition. For instance, Rodríguez-Rodríguez et al. [27] used anthropometric measurements. Three others [24, 99, 118] used BIA, three [26, 28, 33] used ADP, and eight [25, 29–32, 34, 98, 133] used DXA. The different used techniques could explain the differences in the results of fat mass and subsequently of lean mass, since, as shown in other studies [153, 154], these body composition methods are not interchangeable and can affect the results due to intra-instrument and inter-instrument variability. Factors related to the technician (that is, intra-operator and inter-operator variability), factors related to the subject (that is, preparation of the subject as position and measurement schedule, among others) and even factors related to the environment (for example the temperature of the environment in the case of BIA) will influence results [137].

Thirdly, some studies [24–31, 33, 98, 133] used absolute or relative FFM/LBM measures (kg and %), which makes it difficult to compare the individuals of different sizes adequately, given that FFM varies with height, weight, and age, and FFM percentage decreases automatically in proportion to increases in % body fat. Some studies [32, 34, 118] did show different indices using measures adjusted for height (kg/m^2^) or lean to fat ratio (muscle mass (kg)/fat mass (kg) [24].

Furthermore, using absolute (kg) or relative values (% or index) can generate different and even contradictory results. For instance, in the study developed by Masquio et al. [26], the MetS group presented a significantly higher FFM (kg); however, this difference was not significant when the analysis was performed as a percentage. In the study conducted by Ayvaz et al. [118], there were no differences reported in FFM (kg) and FFM (%) between participants with MetS. However, when the results were presented as FFMI, it was found that children with obesity and MetS had lower FFMI values than those without MetS.

Other authors have recently also highlighted that the way of expressing FFM/LBM (absolute vs. relative values) greatly influences the direction of the association with metabolic health [150]. Further studies considering height adjusted indices to assess FFM/LBM are necessary [155].

Fourth, the selection bias of the patients who participated in the included studies may have influenced the present meta-analysis results. Besides, the publication bias could potentially have led to an underestimation of the pooled estimates.

Fifth, most of the studies included in this review did not investigate the association between FFM/LBM and IR/GT/MetS in children and adolescents as their primary objective, limiting the results presented here as they are not studies especially designed for this.

Sixth, this review did not include grey literature such as technical reports, conference proceedings, and doctoral theses, which could also prove helpful.

Seventh, in the different analysis, very few studies evaluated their effect; therefore, the results should be viewed with caution.

Eight, high heterogeneity was found in some of the performed meta-analysis. This could be due to the fact that we could not control the possible covariates (population enrolled, study design, methodologies for determining body composition and glucose homeostasis, maturation, nutritional status) that may explain this heterogeneity because of the low number of studies included, calling for caution in the interpretation of the results.

Lastly, the strength of the evidence is low due to the observational design of almost all included studies, and it was not possible to establish any causal relationship between FFM/LBM and IR/GT/MetS in children/adolescents. The certainty of the evidence was reduced to low and very low, mainly due to the inconsistency and imprecision of the included studies.

However, this study also has several strengths. As far as we know, this study is the first systematic review and meta-analysis that examines the differences in FFM/LBM according to the presence of IR/GT/MetS in children and adolescents. This review followed strict procedures to ensure the validity of the results (registered in the PROSPERO database, PRISMA protocol, two reviewers, quality evaluation of the studies, use of the GRADE system to rate the certainty of the evidence, the performance of a meta-analysis).

Based on the findings of this review, there are a number of considerations for future research in this area. It is necessary to define a criterion for the classification of MetS in children, given the different existing criteria in the literature. In addition, an attempt should be made to unify a unit of expression of the FFM/LBM since different expressions can lead to contrasting conclusions.

Finally, research studies should focus on explaining the effect of FFM/LBM on different metabolic outcomes, preferably involving representative population samples and robust body composition techniques to obtain a better understanding of such associations. Moreover, it may be interesting to evaluate the quality (muscle density), composition (accumulation of fat inside the muscle or intramuscular adipose tissue), or the functional results (relative strength) in addition to the absolute (kg) and relative (%) values in future studies. Additionally, further studies should evaluate those factors that during the prenatal period and early postnatal development can affect the results of FFM/LBM.

## Conclusion

The main finding of this systematic review is that there is limited evidence on the impact of FFM/LBM on IS/IR/GT/MetS in children and adolescents, and the available literature is contradictory. Furthermore, the way of expressing FFM/LBM influences the observed results on its association with IS/IR/GT/MetS. Our results indicate a lower percentage of FFM/LBM in participants with IR/GT/MetS. At the same time, higher values were found when expressed in mass (kg) units.

This research proposes a new study scenario that considers the effect of FFM/LBM on metabolic outcomes to explain the inconsistent association with obesity assessed using the BMI. This reinforces the routine assessing body composition in the pediatric population.

## Supplementary Information

**Additional file 1: Table S1.** Search strategy for systematic reviews and systematic review protocols. **Table S2.** PRISMA-S Checklist. **Table S3*.*** Quality assessment of the included cross-sectional studies. **Table S4.** Quality assessment of the included longitudinal study. **Table S5.** Quality assessment of the included clinical trial. **Table S6.** Grading of Recommendations, Assessment, Development, and Evaluation (GRADE) summary of findings. **Table S7.** PRISMA 2020 for abstracts Checklist.

## Data Availability

All data generated or analyzed during this study are included in this published article [and its supplementary information files].

## Abbreviations

IR: Insulin resistance
IS: Insulin sensitivity
MetS: Metabolic syndrome
BP: Blood pressure
TG: Triglycerides
HDL-C: HDL cholesterol
FPG: Fasting plasma glucose
BMI: Body mass index
FM: Fat mass
FFM: Fat-free mass
LBM: Lean body mass
GT: Glucose tolerance
FFMI: Fat-free mass index
HOMA-IR: Homeostasis model assessment-insulin resistance
