# Supplementary material for: Fat-free/lean body mass in children with insulin resistance or metabolic syndrome: a systematic review and meta-analysis
Source: BMC Pediatr. 2022 Jan 22;22:58. doi: 10.1186/s12887-021-03041-z (PMC8783460; doi:10.1186/s12887-021-03041-z)
Supplement: Supplementary file 1 — Additional file 1: Table S1. Search strategy for systematic reviews and systematic review protocols. Table S2. PRISMA-S Checklist. Table S3. Quality assessment of the included cross-sectional studies. Table S4. Quality assessment of the included longitudinal study. Table S5. Quality assessment of the included clinical trial. Table S6. Grading of Recommendations, Assessment, Development, and Evaluation (GRADE) summary of findings. Table S7. PRISMA 2020 for abstracts Checklist. [file 12887_2021_3041_MOESM1_ESM.zip › Table S1.docx]

## Fat-free /lean body mass in children with insulin resistance or metabolic syndrome: a systematic review and meta-analysis

Diana Paola Córdoba-Rodríguez^1^, Iris Iglesia ^2,3,4^, Alejandro Gomez-Bruton ^2,5,6^, Gerardo Rodríguez^2,3,4,6,7^, José Antonio Casajús^2,5^, Hernan Morales-Devia^8^, Luis A. Moreno^2,4,6^.

1 Departamento de Nutrición y Bioquímica, Facultad de Ciencias, Pontificia Universidad Javeriana, Bogotá DC, Colombia.

2 Growth, Exercise, Nutrition and Development (GENUD) Research Group, Universidad de Zaragoza.

3 Instituto Agroalimentario de Aragón (IA2), Instituto de Investigación Sanitaria Aragón (IIS Aragón), Zaragoza, España

4 Red de Salud Materno Infantil y del Desarrollo (SAMID), Instituto de Salud Carlos III,Madrid,España.

5 Faculty of Health and Sport Sciences (FCSD), Department of Physiatry and Nursing, University of Zaragoza, Spain.

6 Centro de Investigación Biomédica en Red de Fisiopatología de la Obesidad y Nutrición (CIBERObn), Instituto de Salud Carlos III, Madrid, Spain.

7 Departamento de Pediatría, Universidad de Zaragoza, Zaragoza, España.

8 Biblioteca General Alfonso Borrero Cabal, Pontificia Universidad Javeriana, Bogotá, Colombia

Corresponding author: Alejandro Gomez-Bruton

E-mail: [bruton@unizar.es](mailto:bruton@unizar.es) https://orcid.org/0000-0002-0520-1640

Diana Paola Córdoba Rodríguez: [d.cordoba@javeriana.edu.co](mailto:d.cordoba@javeriana.edu.co) https://orcid.org/0000-0002-7034-8796

Iris Iglesia: [iglesia@unizar.es](mailto:iglesia@unizar.es) https://orcid.org/0000-0002-2219-3646

Alejandro Gómez Bruton: [bruton@unizar.es](mailto:bruton@unizar.es) <https://orcid.org/0000-0002-0520-1640>

Gerardo Rodríguez-Martínez: [gerard@unizar.es](mailto:gerard@unizar.es) https://orcid.org/0000-0002-7985-9912

José Antonio Casajús: [joseant@unizar.es](mailto:joseant@unizar.es) https://orcid.org/0000-0002-7215-6931

Hernan Morales-Devia: [hmorales@javeriana.edu.co](mailto:hmorales@javeriana.edu.co) https://orcid.org/0000-0002-8895-7864

Luis A. Moreno: [lmoreno@unizar.es](mailto:lmoreno@unizar.es) https://orcid.org/0000-0003-0454-653X

**Table S1. Search strategy for systematic reviews and systematic review protocols.**

| Database | Search Query |
| --- | --- |
| PubMed  (Search date: 21/06/21; 2632 results) | (("Metabolic Syndrome"[Title/Abstract] OR "Metabolic Syndrome"[MeSH Terms] OR "Insulin Resistance"[Title/Abstract] OR "Insulin Resistance"[MeSH Terms] OR "insulin sensitive"[Title/Abstract] OR "insulin sensitivity"[Title/Abstract] OR "glucose tolerance"[Title/Abstract]) AND ("Body Composition"[Title/Abstract] OR "Body Composition"[MeSH Terms] OR "lean body mass"[Title/Abstract] OR "fat free mass"[Title/Abstract] OR "lean mass"[Title/Abstract] OR "lean tissue mass"[Title/Abstract] OR "skeletal muscle mass"[Title/Abstract] OR "muscle mass"[Title/Abstract] OR "fat free mass index"[Title/Abstract]) AND ("infant*"[Title/Abstract] OR "infant"[MeSH Terms] OR "child*"[Title/Abstract] OR "child"[MeSH Terms] OR "adolescen*"[Title/Abstract] OR "adolescent"[MeSH Terms])) AND ((humans[Filter]) AND (1970:2021[pdat])) |
| Embase  (Search date: 21/06/21; 4190 results) | ('metabolic syndrome':ti,ab,kw OR 'metabolic syndrome x':ti,ab,kw OR 'metabolic syndrome x'/exp OR 'insulin sensitive':ti,ab,kw OR 'insulin sensitivity':ti,ab,kw OR 'insulin sensitivity'/exp OR 'insulin resistance':ti,ab,kw OR 'insulin resistance'/exp OR 'glucose tolerance'/exp OR 'glucose tolerance':ti,ab,kw) AND ('body composition':ti,ab,kw OR 'body composition'/exp OR 'lean body mass':ti,ab,kw OR 'lean body weight':ti,ab,kw OR 'lean body weight'/exp OR 'fat free mass':ti,ab,kw OR 'fat free mass'/exp OR 'fat free mass index':ti,ab,kw OR 'fat free mass index'/exp OR 'lean mass':ti,ab,kw OR 'lean mass'/exp OR 'lean tissue mass':ti,ab,kw OR 'lean tissue mass'/exp OR 'skeletal muscle mass':ti,ab,kw OR 'skeletal muscle mass'/exp OR 'skeletal muscle mass index':ti,ab,kw OR 'skeletal muscle mass index'/exp OR 'muscle mass':ti,ab,kw OR 'muscle mass'/exp OR 'muscle mass index':ti,ab,kw OR 'muscle mass index'/exp) AND ('infant*':ti,ab,kw OR 'infant'/exp OR 'child*':ti,ab,kw OR 'child'/exp OR 'adolescen*':ti,ab,kw OR 'adolescence'/exp OR 'adolescent'/exp) AND [humans]/lim AND [<1966-2021]/py |
| Scopus  (Search date: 21/06/21; 3140 results) | TITLE-ABS-KEY(("Metabolic Syndrome" OR "Insulin Resistance" OR "insulin sensitiv*" OR "glucose tolerance") AND ("Body Composition" OR "lean body mass" OR "lean body weight" OR "fat free mass" OR "lean mass" OR "lean tissue mass" OR "skeletal muscle mass" OR "muscle mass" OR "fat free mass index") AND (infant* OR child* OR adolescen*)) PUBYEAR < 2022 |
| Web of Science  Core Collection (2001-present)  (Search date: 21/06/21; 2678 results) | TS=(("Metabolic Syndrome" OR "Insulin Resistance" OR "insulin sensitiv*" OR "glucose tolerance") AND ("Body Composition" OR "lean body mass" OR "lean body weight" OR "fat free mass" OR "lean mass" OR "lean tissue mass" OR "skeletal muscle mass" OR "muscle mass" OR "fat free mass index") AND (infant* OR child* OR adolescen*) ) AND PY=(2001-2021) |
| sciELO.org  (Search date: 21/06/21; 4 results) | subject:(("Metabolic Syndrome" OR "Insulin Resistance" OR "insulin sensitiv*" OR “glucose tolerance”) AND ("Body Composition" OR "lean body mass" OR "lean body weight" OR "fat free mass" OR "lean mass" OR "lean tissue mass" OR "skeletal muscle mass" OR "muscle mass" OR "fat free mass index") AND (infant* OR child* OR adolescen*)) |
