# Supplementary material for: Fat-free/lean body mass in children with insulin resistance or metabolic syndrome: a systematic review and meta-analysis
Source: BMC Pediatr. 2022 Jan 22;22:58. doi: 10.1186/s12887-021-03041-z (PMC8783460; doi:10.1186/s12887-021-03041-z)
Supplement: Supplementary file 1 — Additional file 1: Table S1. Search strategy for systematic reviews and systematic review protocols. Table S2. PRISMA-S Checklist. Table S3. Quality assessment of the included cross-sectional studies. Table S4. Quality assessment of the included longitudinal study. Table S5. Quality assessment of the included clinical trial. Table S6. Grading of Recommendations, Assessment, Development, and Evaluation (GRADE) summary of findings. Table S7. PRISMA 2020 for abstracts Checklist. [file 12887_2021_3041_MOESM1_ESM.zip › Table S2.docx]

## Fat-free /lean body mass in children with insulin resistance or metabolic syndrome: a systematic review and meta-analysis

Diana Paola Córdoba-Rodríguez^1^, Iris Iglesia ^2,3,4^, Alejandro Gomez-Bruton ^2,5,6^, Gerardo Rodríguez^2,3,4,6,7^, José Antonio Casajús^2,5^, Hernan Morales-Devia^8^, Luis A. Moreno^2,4,6^.

1 Departamento de Nutrición y Bioquímica, Facultad de Ciencias, Pontificia Universidad Javeriana, Bogotá DC, Colombia.

2 Growth, Exercise, Nutrition and Development (GENUD) Research Group, Universidad de Zaragoza.

3 Instituto Agroalimentario de Aragón (IA2), Instituto de Investigación Sanitaria Aragón (IIS Aragón), Zaragoza, España

4 Red de Salud Materno Infantil y del Desarrollo (SAMID), Instituto de Salud Carlos III,Madrid,España.

5 Faculty of Health and Sport Sciences (FCSD), Department of Physiatry and Nursing, University of Zaragoza, Spain.

6 Centro de Investigación Biomédica en Red de Fisiopatología de la Obesidad y Nutrición (CIBERObn), Instituto de Salud Carlos III, Madrid, Spain.

7 Departamento de Pediatría, Universidad de Zaragoza, Zaragoza, España.

8 Biblioteca General Alfonso Borrero Cabal, Pontificia Universidad Javeriana, Bogotá, Colombia

Corresponding author: Alejandro Gomez-Bruton

E-mail: [bruton@unizar.es](mailto:bruton@unizar.es) https://orcid.org/0000-0002-0520-1640

Diana Paola Córdoba Rodríguez: [d.cordoba@javeriana.edu.co](mailto:d.cordoba@javeriana.edu.co) https://orcid.org/0000-0002-7034-8796

Iris Iglesia: [iglesia@unizar.es](mailto:iglesia@unizar.es) https://orcid.org/0000-0002-2219-3646

Alejandro Gómez Bruton: [bruton@unizar.es](mailto:bruton@unizar.es) <https://orcid.org/0000-0002-0520-1640>

Gerardo Rodríguez-Martínez: [gerard@unizar.es](mailto:gerard@unizar.es) https://orcid.org/0000-0002-7985-9912

José Antonio Casajús: [joseant@unizar.es](mailto:joseant@unizar.es) https://orcid.org/0000-0002-7215-6931

Hernan Morales-Devia: [hmorales@javeriana.edu.co](mailto:hmorales@javeriana.edu.co) https://orcid.org/0000-0002-8895-7864

Luis A. Moreno: [lmoreno@unizar.es](mailto:lmoreno@unizar.es) https://orcid.org/0000-0003-0454-653X

**Table S2. PRISMA-S Checklist**

| **Section/topic** | **#** | **Checklist item** | **Location(s) Reported** |
| --- | --- | --- | --- |
| **INFORMATION SOURCES AND METHODS** | | | |
| Database name | 1 | Name each individual database searched, stating the platform for each. | Page 7 |
| Multi-database searching | 2 | If databases were searched simultaneously on a single platform, state the name of the platform, listing all of the databases searched. | Page 7 |
| Study registries | 3 | List any study registries searched. | N/A |
| Online resources and browsing | 4 | Describe any online or print source purposefully searched or browsed (e.g., tables of contents, print conference proceedings, web sites), and how this was done. | N/A |
| Citation searching | 5 | Indicate whether cited references or citing references were examined, and describe any methods used for locating cited/citing references (e.g., browsing reference lists, using a citation index, setting up email alerts for references citing included studies). | Page 8- Figure 1 |
| Contacts | 6 | Indicate whether additional studies or data were sought by contacting authors, experts, manufacturers, or others. | Page 10 |
| Other methods | 7 | Describe any additional information sources or search methods used. | N/A |
| **SEARCH STRATEGIES** | | | |
| Full search strategies | 8 | Include the search strategies for each database and information source, copied and pasted exactly as run. | Table S1 |
| Limits and restrictions | 9 | Specify that no limits were used, or describe any limits or restrictions applied to a search (e.g., date or time period, language, study design) and provide justification for their use. | Page 9 |
| Search filters | 10 | Indicate whether published search filters were used (as originally designed or modified), and if so, cite the filter(s) used. | Page 8 |
| Prior work | 11 | Indicate when search strategies from other literature reviews were adapted or reused for a substantive part or all of the search, citing the previous review(s). | N/A |
| Updates | 12 | Report the methods used to update the search(es) (e.g., rerunning searches, email alerts). | 9 |
| Dates of searches | 13 | For each search strategy, provide the date when the last search occurred. | Table S1 |
| **PEER REVIEW** | | | |
| Peer review | 14 | Describe any search peer review process. | Page 8 |
| **MANAGING RECORDS** | | | |
| Total Records | 15 | Document the total number of records identified from each database and other information sources. | Table S1 |
| Deduplication | 16 | Describe the processes and any software used to deduplicate records from multiple database searches and other information sources. | Pages 9-10 |
|  |  |  |  |
| PRISMA-S: An Extension to the PRISMA Statement for Reporting Literature Searches in Systematic Reviews | | |  |
| Rethlefsen ML, Kirtley S, Waffenschmidt S, Ayala AP, Moher D, Page MJ, Koffel JB, PRISMA-S Group. | | |  |
| Last updated February 27, 2020. | |  |  |
