# Supplementary material for: Fat-free/lean body mass in children with insulin resistance or metabolic syndrome: a systematic review and meta-analysis
Source: BMC Pediatr. 2022 Jan 22;22:58. doi: 10.1186/s12887-021-03041-z (PMC8783460; doi:10.1186/s12887-021-03041-z)
Supplement: Supplementary file 1 — Additional file 1: Table S1. Search strategy for systematic reviews and systematic review protocols. Table S2. PRISMA-S Checklist. Table S3. Quality assessment of the included cross-sectional studies. Table S4. Quality assessment of the included longitudinal study. Table S5. Quality assessment of the included clinical trial. Table S6. Grading of Recommendations, Assessment, Development, and Evaluation (GRADE) summary of findings. Table S7. PRISMA 2020 for abstracts Checklist. [file 12887_2021_3041_MOESM1_ESM.zip › Table S6.docx]

**Fat-free /lean body mass in children with insulin resistance or metabolic syndrome: a systematic review and meta-analysis**

Diana Paola Córdoba-Rodríguez^1^, Iris Iglesia ^2,3,4^, Alejandro Gomez-Bruton ^2,5,6^, Gerardo Rodríguez^2,3,4,6,7^, José Antonio Casajús^2,5^, Hernan Morales-Devia^8^, Luis A. Moreno^2,4,6^.

1 Departamento de Nutrición y Bioquímica, Facultad de Ciencias, Pontificia Universidad Javeriana, Bogotá DC, Colombia.

2 Growth, Exercise, Nutrition and Development (GENUD) Research Group, Universidad de Zaragoza.

3 Instituto Agroalimentario de Aragón (IA2), Instituto de Investigación Sanitaria Aragón (IIS Aragón), Zaragoza, España

4 Red de Salud Materno Infantil y del Desarrollo (SAMID), Instituto de Salud Carlos III,Madrid,España.

5 Faculty of Health and Sport Sciences (FCSD), Department of Physiatry and Nursing, University of Zaragoza, Spain.

6 Centro de Investigación Biomédica en Red de Fisiopatología de la Obesidad y Nutrición (CIBERObn), Instituto de Salud Carlos III, Madrid, Spain.

7 Departamento de Pediatría, Universidad de Zaragoza, Zaragoza, España.

8 Biblioteca General Alfonso Borrero Cabal, Pontificia Universidad Javeriana, Bogotá, Colombia

Corresponding author: Alejandro Gomez-Bruton

E-mail: [bruton@unizar.es](mailto:bruton@unizar.es) https://orcid.org/0000-0002-0520-1640

Diana Paola Córdoba Rodríguez: [d.cordoba@javeriana.edu.co](mailto:d.cordoba@javeriana.edu.co) https://orcid.org/0000-0002-7034-8796

Iris Iglesia: [iglesia@unizar.es](mailto:iglesia@unizar.es) https://orcid.org/0000-0002-2219-3646

Alejandro Gómez Bruton: [bruton@unizar.es](mailto:bruton@unizar.es) <https://orcid.org/0000-0002-0520-1640>

Gerardo Rodríguez-Martínez: [gerard@unizar.es](mailto:gerard@unizar.es) https://orcid.org/0000-0002-7985-9912

José Antonio Casajús: [joseant@unizar.es](mailto:joseant@unizar.es) https://orcid.org/0000-0002-7215-6931

Hernan Morales-Devia: [hmorales@javeriana.edu.co](mailto:hmorales@javeriana.edu.co) https://orcid.org/0000-0002-8895-7864

Luis A. Moreno: [lmoreno@unizar.es](mailto:lmoreno@unizar.es) https://orcid.org/0000-0003-0454-653X

**Table S6 Grading of Recommendations, Assessment, Development, and Evaluation (GRADE) summary of findings**

| **Certainty assessment** | | | | | | | **№ of patients** | | **Effect** | | **Certainty** | **Importance** |
| --- | --- | --- | --- | --- | --- | --- | --- | --- | --- | --- | --- | --- |
| **№ of studies** | **Study design** | **Risk of bias** | **Inconsistency** | **Indirectness** | **Imprecision** | **Other considerations** | **with IR/GT/MetS** | **without IR/GT/MetS** | **Relative (95% CI)** | **Absolute (95% CI)** |  |  |
| **FFM / LBM (%)** | | | | | | | | | | | | |
| 7 | observational studies | not serious | very serious^a^ | not serious | very serious^b^ | strong association | 236 | 1230 | - | SMD **0.47 SD lower** (0.62 lower to 0.32 lower) | ⨁◯◯◯ Very low | CRITICAL |
| **FFM / LBM (%): Subgroup analyses by diagnosis (group IR and group MetS)** | | | | | | | | | | | | |
| 7 | observational studies | not serious | very serious^c^ | not serious | very serious^d^ | none | 236 | 1230 | - | SMD **0.47 SD lower** (0.62 lower to 0.32 lower) | ⨁◯◯◯ Very low | IMPORTANT |
| **FFM / LBM (kg)** | | | | | | | | | | | | |
| 12 | observational studies | not serious | very serious^e^ | not serious | not serious | none | 356 | 614 | - | SMD **1.01 SD higher** (0.43 higher to 1.6 higher) | ⨁⨁◯◯ Low | CRITICAL |
| **FFM / LBM (kg): Subgroup analyses by diagnosis (group GT and group MetS)** | | | | | | | | | | | | |
| 11 | observational studies | not serious | very serious^f^ | not serious | not serious | none | 317 | 587 | - | SMD **1.05 SD higher** (0.41 higher to 1.69 higher) | ⨁⨁◯◯ Low | IMPORTANT |
| **FFM / LBM (kg): Subgroup analysis by a device (anthropometric measurements and BIA group and BOD-POD and DXA group)** | | | | | | | | | | | | |
| 11 | observational studies | not serious | very serious^g^ | not serious | not serious | none | 317 | 587 | - | SMD **1.05 SD higher** (0.41 higher to 1.69 higher) | ⨁⨁◯◯ Low | IMPORTANT |

**CI:** confidence interval; **SMD:** standardised mean difference

#### Explanations

a. The heterogeneity between the studies was moderate (I2 = 73; p = 0.001).

b. Wide IC of the measure of effect.

c. The heterogeneity between the studies was moderate (I2 = 73; p = 0.001).

d. Wide IC of the measure of effect.

e. The heterogeneity was high (I2 = 93; p = <0.001).

f. The heterogeneity was high (I2 = 98; p< 0.01).

g. The heterogeneity was high (I2 = 97; p< 0.01).
