# Supplementary material for: Fat-free/lean body mass in children with insulin resistance or metabolic syndrome: a systematic review and meta-analysis
Source: BMC Pediatr. 2022 Jan 22;22:58. doi: 10.1186/s12887-021-03041-z (PMC8783460; doi:10.1186/s12887-021-03041-z)
Supplement: Supplementary file 1 — Additional file 1: Table S1. Search strategy for systematic reviews and systematic review protocols. Table S2. PRISMA-S Checklist. Table S3. Quality assessment of the included cross-sectional studies. Table S4. Quality assessment of the included longitudinal study. Table S5. Quality assessment of the included clinical trial. Table S6. Grading of Recommendations, Assessment, Development, and Evaluation (GRADE) summary of findings. Table S7. PRISMA 2020 for abstracts Checklist. [file 12887_2021_3041_MOESM1_ESM.zip › Table S7.docx]

**Table S7 PRISMA 2020 for abstracts Checklist**.

*From:*  Page MJ, McKenzie JE, Bossuyt PM, Boutron I, Hoffmann TC, Mulrow CD, et al. The PRISMA 2020 statement: an updated guideline for reporting systematic reviews. BMJ 2021;372:n71. doi: 10.1136/bmj.n71

| **Section and Topic** | **Item #** | **Checklist item** | **Reported (Yes/No)** |
| --- | --- | --- | --- |
| **TITLE** | | |  |
| Title | 1 | Identify the report as a systematic review. | Yes |
| **BACKGROUND** | | |  |
| Objectives | 2 | Provide an explicit statement of the main objective(s) or question(s) the review addresses. | Yes |
| **METHODS** | | |  |
| Eligibility criteria | 3 | Specify the inclusion and exclusion criteria for the review. | Yes |
| Information sources | 4 | Specify the information sources (e.g. databases, registers) used to identify studies and the date when each was last searched. | Yes |
| Risk of bias | 5 | Specify the methods used to assess risk of bias in the included studies. | Yes |
| Synthesis of results | 6 | Specify the methods used to present and synthesise results. | Yes |
| **RESULTS** | | |  |
| Included studies | 7 | Give the total number of included studies and participants and summarise relevant characteristics of studies. | Yes |
| Synthesis of results | 8 | Present results for main outcomes, preferably indicating the number of included studies and participants for each. If meta-analysis was done, report the summary estimate and confidence/credible interval. If comparing groups, indicate the direction of the effect (i.e. which group is favoured). | Yes |
| **DISCUSSION** | | |  |
| Limitations of evidence | 9 | Provide a brief summary of the limitations of the evidence included in the review (e.g. study risk of bias, inconsistency and imprecision). | Yes |
| Interpretation | 10 | Provide a general interpretation of the results and important implications. | Yes |
| **OTHER** | | |  |
| Funding | 11 | Specify the primary source of funding for the review. | NA |
| Registration | 12 | Provide the register name and registration number. | Yes |

For more information, visit: <http://www.prisma-statement.org/>
