# Supplementary material for: Fat-free/lean body mass in children with insulin resistance or metabolic syndrome: a systematic review and meta-analysis
Source: BMC Pediatr. 2022 Jan 22;22:58. doi: 10.1186/s12887-021-03041-z (PMC8783460; doi:10.1186/s12887-021-03041-z)
Supplement: Supplementary file 1 — Additional file 1: Table S1. Search strategy for systematic reviews and systematic review protocols. Table S2. PRISMA-S Checklist. Table S3. Quality assessment of the included cross-sectional studies. Table S4. Quality assessment of the included longitudinal study. Table S5. Quality assessment of the included clinical trial. Table S6. Grading of Recommendations, Assessment, Development, and Evaluation (GRADE) summary of findings. Table S7. PRISMA 2020 for abstracts Checklist. [file 12887_2021_3041_MOESM1_ESM.zip › Table S3.doc]

## Fat-free /lean body mass in children with insulin resistance or metabolic syndrome: a systematic review and meta-analysis

Diana Paola Córdoba-Rodríguez^1^, Iris Iglesia ^2,3,4^, Alejandro Gomez-Bruton ^2,5,6^, Gerardo Rodríguez^2,3,4,6,7^, José Antonio Casajús^2,5^, Hernan Morales-Devia^8^, Luis A. Moreno^2,4,6^.

1 Departamento de Nutrición y Bioquímica, Facultad de Ciencias, Pontificia Universidad Javeriana, Bogotá DC, Colombia.

2 Growth, Exercise, Nutrition and Development (GENUD) Research Group, Universidad de Zaragoza.

3 Instituto Agroalimentario de Aragón (IA2), Instituto de Investigación Sanitaria Aragón (IIS Aragón), Zaragoza, España

4 Red de Salud Materno Infantil y del Desarrollo (SAMID), Instituto de Salud Carlos III,Madrid,España.

5 Faculty of Health and Sport Sciences (FCSD), Department of Physiatry and Nursing, University of Zaragoza, Spain.

6 Centro de Investigación Biomédica en Red de Fisiopatología de la Obesidad y Nutrición (CIBERObn), Instituto de Salud Carlos III, Madrid, Spain.

7 Departamento de Pediatría, Universidad de Zaragoza, Zaragoza, España.

8 Biblioteca General Alfonso Borrero Cabal, Pontificia Universidad Javeriana, Bogotá, Colombia

Corresponding author: Alejandro Gomez-Bruton

E-mail: [bruton@unizar.es](mailto:bruton@unizar.es) https://orcid.org/0000-0002-0520-1640

Diana Paola Córdoba Rodríguez: [d.cordoba@javeriana.edu.co](mailto:d.cordoba@javeriana.edu.co) https://orcid.org/0000-0002-7034-8796

Iris Iglesia: [iglesia@unizar.es](mailto:iglesia@unizar.es) https://orcid.org/0000-0002-2219-3646

Alejandro Gómez Bruton: [bruton@unizar.es](mailto:bruton@unizar.es) <https://orcid.org/0000-0002-0520-1640>

Gerardo Rodríguez-Martínez: [gerard@unizar.es](mailto:gerard@unizar.es) https://orcid.org/0000-0002-7985-9912

José Antonio Casajús: [joseant@unizar.es](mailto:joseant@unizar.es) https://orcid.org/0000-0002-7215-6931

Hernan Morales-Devia: [hmorales@javeriana.edu.co](mailto:hmorales@javeriana.edu.co) https://orcid.org/0000-0002-8895-7864

Luis A. Moreno: [lmoreno@unizar.es](mailto:lmoreno@unizar.es) https://orcid.org/0000-0003-0454-653X

**Table S3 Quality assessment of the included cross-sectional studies**.

| **Cross-sectional studies** | **Appropriate Research Design? Y/N** | **Appropriate Recruitment Strategy? Y/N** | **¿Response rate? % Y/N** | **Is the sample representative? Y/N** | **Objective and reliable measures? Y/N** | **Power calculation/ Justification numbers? Y/N** | **Appropriate Statistical Analyses? Y/N** | **QUALITY INDICATORS MET (TOTAL): 0-2 LOW; 3-5 MODERATE; 6-7 HIGH** |
| --- | --- | --- | --- | --- | --- | --- | --- | --- |
| Behrooz et al. [100] | Y | Y | N | N | Y | Y | Y | 5 Moderate |
| Kim, et al. [25] | Y | Y | N | N | Y | N | Y | 4 Moderate |
| Gonzalez‑Gil et al. [24] | Y | Y | N | N | Y | N | Y | 4 Moderate |
| Burrows et al. [30] | Y | Y | N | N | Y | N | Y | 4 Moderate |
| Weber et al. [26] | Y | Y | N | Y | Y | N | Y | 5 Moderate |
| Rodríguez-Rodríguez et al. [28] | Y | Y | N | N | Y | N | Y | 4 Moderate |
| Ayvaz et al. [119] | Y | Y | N | N | Y | N | Y | 4 Moderate |
| Brufani et al. [33] | Y | Y | N | Y | Y | N | Y | 5 Moderate |
| Hsu et al. [34] | Y | Y | N | N | Y | N | Y | 4 Moderate |
| Goran et al. [99] | Y | Y | N | N | Y | N | Y | 4 Moderate |
| Weiss et al. [31] | Y | Y | N | Y | Y | Y | Y | 6 High |

Y=Yes; N=No. Checklist criteria from: “Criteria for the evaluation of qualitative research papers” [36]
