# Supplementary material for: Fat-free/lean body mass in children with insulin resistance or metabolic syndrome: a systematic review and meta-analysis
Source: BMC Pediatr. 2022 Jan 22;22:58. doi: 10.1186/s12887-021-03041-z (PMC8783460; doi:10.1186/s12887-021-03041-z)
Supplement: Supplementary file 1 — Additional file 1: Table S1. Search strategy for systematic reviews and systematic review protocols. Table S2. PRISMA-S Checklist. Table S3. Quality assessment of the included cross-sectional studies. Table S4. Quality assessment of the included longitudinal study. Table S5. Quality assessment of the included clinical trial. Table S6. Grading of Recommendations, Assessment, Development, and Evaluation (GRADE) summary of findings. Table S7. PRISMA 2020 for abstracts Checklist. [file 12887_2021_3041_MOESM1_ESM.zip › Table S4.doc]

## Fat-free /lean body mass in children with insulin resistance or metabolic syndrome: a systematic review and meta-analysis

Diana Paola Córdoba-Rodríguez^1^, Iris Iglesia ^2,3,4^, Alejandro Gomez-Bruton ^2,5,6^, Gerardo Rodríguez^2,3,4,6,7^, José Antonio Casajús^2,5^, Hernan Morales-Devia^8^, Luis A. Moreno^2,4,6^.

1 Departamento de Nutrición y Bioquímica, Facultad de Ciencias, Pontificia Universidad Javeriana, Bogotá DC, Colombia.

2 Growth, Exercise, Nutrition and Development (GENUD) Research Group, Universidad de Zaragoza.

3 Instituto Agroalimentario de Aragón (IA2), Instituto de Investigación Sanitaria Aragón (IIS Aragón), Zaragoza, España

4 Red de Salud Materno Infantil y del Desarrollo (SAMID), Instituto de Salud Carlos III,Madrid,España.

5 Faculty of Health and Sport Sciences (FCSD), Department of Physiatry and Nursing, University of Zaragoza, Spain.

6 Centro de Investigación Biomédica en Red de Fisiopatología de la Obesidad y Nutrición (CIBERObn), Instituto de Salud Carlos III, Madrid, Spain.

7 Departamento de Pediatría, Universidad de Zaragoza, Zaragoza, España.

8 Biblioteca General Alfonso Borrero Cabal, Pontificia Universidad Javeriana, Bogotá, Colombia

Corresponding author: Alejandro Gomez-Bruton

E-mail: [bruton@unizar.es](mailto:bruton@unizar.es) https://orcid.org/0000-0002-0520-1640

Diana Paola Córdoba Rodríguez: [d.cordoba@javeriana.edu.co](mailto:d.cordoba@javeriana.edu.co) https://orcid.org/0000-0002-7034-8796

Iris Iglesia: [iglesia@unizar.es](mailto:iglesia@unizar.es) https://orcid.org/0000-0002-2219-3646

Alejandro Gómez Bruton: [bruton@unizar.es](mailto:bruton@unizar.es) <https://orcid.org/0000-0002-0520-1640>

Gerardo Rodríguez-Martínez: [gerard@unizar.es](mailto:gerard@unizar.es) https://orcid.org/0000-0002-7985-9912

José Antonio Casajús: [joseant@unizar.es](mailto:joseant@unizar.es) https://orcid.org/0000-0002-7215-6931

Hernan Morales-Devia: [hmorales@javeriana.edu.co](mailto:hmorales@javeriana.edu.co) https://orcid.org/0000-0002-8895-7864

Luis A. Moreno: [lmoreno@unizar.es](mailto:lmoreno@unizar.es) https://orcid.org/0000-0003-0454-653X

**Table S4 Quality assessment of the included longitudinal study.**

| **Studies** |  | **Criterion** | **Score** |
| --- | --- | --- | --- |
| Brufani et al. [32] | 1 | Are the objectives or hypotheses of the study started? | Y |
|  | 2 | Is the target population defined? | Y |
|  | 3 | Is the sampling frame defined? | Y |
|  | 4 | Is the study population defined? | Y |
|  | 5 | Are the study setting (venues) and/or geographic location stated? | Y |
|  | 6 | Are the dates between which the study was conducted started or implicit? | Y |
|  | 7 | Are eligibility criteria started? | Y |
|  | 8 | Are issues of ¨selection¨ to the study mentioned? | Y |
|  | 9 | Is the number of participants justified? | N |
|  | 10 | Are numbers meeting and not meeting the eligibility criteria stated? | N |
|  | 11 | For those not eligible, are the reasons why stated? | N |
|  | 12 | Are the numbers of people who did/did not consent to participate stated? | N |
|  | 13 | Are the reasons that people refused to consent stated? | N |
|  | 14 | Were consenters compared with nonconsenters? | N |
|  | 15 | Was the number of participants at the beginning of the study stated? | N |
|  | 16 | Were methods of data collection stated? | Y |
|  | 17 | Was the reliability (repeatability) of measurement methods mentioned? | Y |
|  | 18 | Was the validity (against a “gold standard”) of measurement methods mentioned? | N |
|  | 19 | Were any confounders mentioned? | N |
|  | 20 | Was the number of participants at each stage/wave specified? | N |
|  | 21 | Were reasons for loss to follow-up quantified? | N |
|  | 22 | Was the missingness of data items at each wave mentioned? | N |
|  | 23 | Was the type of analyses conducted stated? | Y |
|  | 24 | Were “longitudinal” analysis methods stated? | N |
|  | 25 | Were “longitudinal” analysis methods stated? | N |
|  | 26 | Were relative effect sizes reported? | N |
|  | 27 | Was loss to follow-up taken into account in the analysis? | N |
|  | 28 | Were confounders accounted for in analyses? | Y |
|  | 29 | Were missing data accounted for in the analyses? | N |
|  | 30 | Was the impact of biases assessed qualitatively? | N |
|  | 31 | Was the impact of biases assessed qualitatively? | N |
|  | 32 | Did authors relate results back to a target population? | Y |
|  | 33 | Was there any other discussion of generalizability? | Y |
|  |  | **Total** | 13 |

Y=Yes; N=No. Checklist criteria from: “Quality of reporting of observational longitudinal research” [37]
